# Glycyrrhizic Acid Nanoparticles Subside the Activity of Methicillin-Resistant *Staphylococcus aureus* by Suppressing PBP2a

**DOI:** 10.3390/ph17050589

**Published:** 2024-05-06

**Authors:** Patricia Rijo, Tamer M. M. Abuamara, Lashin Saad Ali Lashin, Sherif A. Kamar, Vera M. S. Isca, Tahseen S. Mohammed, Mohamed S. M. Abdrabo, Mohamed A. Amin, Ahmed I. Abd El Maksoud, Amr Hassan

**Affiliations:** 1 CBIOS—Lusófona University’s Research Center for Biosciences and Health Technologies, 1749-024 Lisbon, Portugal; vera.isca@ulusofona.pt; 2 Instituto de Investigação do Medicamento (iMed.ULisboa), Faculdade de Farmácia, Universidade de Lisboa, 1649-003 Lisbon, Portugal; 3 Department of Basic Medical Science, Faculty of Dentistry, Al-Ahliyya Amman University, Amman 19111, Jordan; t.abuamara@ammanu.edu.jo (T.M.M.A.); l.lashin@ammanu.edu.jo (L.S.A.L.); s.qamar@ammanu.edu.jo (S.A.K.); 4 Department of Histology, Faculty of Medicine, Al-Azhar University, Cairo 11884, Egypt; 5 Department of Medical Physiology, Faculty of Medicine, Mansoura University, Mansoura 35516, Egypt; 6 Department of Anatomy and Embryology, Faculty of Medicine, Ain Shams University, Cairo 11566, Egypt; 7 Department of Public Health and Community Medicine, Faculty of Medicine, Al-Azhar University, Cairo 11884, Egypt; drtahseen_55@yahoo.com (T.S.M.); yasssomohsobhy@gmail.com (M.S.M.A.); 8 Department of Basic Medical Science, Faculty of Dentistry, Zarqa University, Zarqa 13110, Jordan; drabdo95@gmail.com; 9 Department of Microbiology and Immunology, Faculty of Medicine, Al-Azhar University, Cairo 11884, Egypt; 10 College of Biotechnology, Misr University of Science and Technology, Giza 12573, Egypt; aiaky79@gmail.com; 11 Department of Industrial Biotechnology, Genetic Engineering and Biotechnology Research Institute (GEBRI), University of Sadat City, Sadat 32897, Egypt; 12 Department of Bioinformatics, Genetic Engineering and Biotechnology Research Institute (GEBRI), University of Sadat City, Sadat 32897, Egypt

**Keywords:** penicillin-binding protein 2a, MRSA, mecA, mecR1, glycyrrhizic acid nanoparticles

## Abstract

*Staphylococcus aureus* and methicillin-resistant *Staphylococcus aureus* (MRSA) are classified as high-risk infections that can lead to death, particularly among older individuals. Nowadays, plant nanoparticles such as glycyrrhizic acid are recognized as efficient bactericides against a wide range of bacterial strains. Recently, scientists have shown interest in plant extract nanoparticles, derived from natural sources, which can be synthesized into nanomaterials. Interestingly, glycyrrhizic acid is rich in antioxidants as well as antibacterial agents, and it exhibits no adverse effects on normal cells. In this study, glycyrrhizic acid nanoparticles (GA-NPs) were synthesized using the hydrothermal method and characterized through physicochemical techniques such as UV–visible spectrometry, DLS, zeta potential, and TEM. The antimicrobial activity of GA-NPs was investigated through various methods, including MIC assays, anti-biofilm activity assays, ATPase activity assays, and kill-time assays. The expression levels of *mecA*, *mecR1*, *blaR1*, and *blaZ* genes were measured by quantitative RT-qPCR. Additionally, the presence of the penicillin-binding protein 2a (PBP2a) protein of *S. aureus* and MRSA was evaluated by a Western blot assay. The results emphasized the fabrication of GA nanoparticles in spherical shapes with a diameter in the range of 40–50 nm. The data show that GA nanoparticles exhibit great bactericidal effectiveness against *S. aureus* and MRSA. The treatment with GA-NPs remarkably reduces the expression levels of the *mecA*, *mecR1*, *blaR1*, and *blaZ* genes. PBP2a expression in MRSA was significantly reduced after treatment with GA-NPs. Overall, this study demonstrates that glycyrrhizic acid nanoparticles have potent antibacterial activity, particularly against MRSA. This research elucidates the inhibition mechanism of glycyrrhizic acid, which involves the suppressing of PBP2a expression. This work emphasizes the importance of utilizing plant nanoparticles as effective antimicrobial agents against a broad spectrum of bacteria.

## 1. Introduction

MRSA is a human pathogen that causes skin infections that can escalate into potentially fatal bacteremia. *S. aureus* and MRSA infections pose serious life-threatening risks. The ability of MRSA to form antibiotic-resistant biofilms contributes to the severity of infections [1]. *S. aureus* is particularly prevalent at the surgical operation site, and its ability to form biofilms can lead to antibiotic resistance [2]. Several bacterial strains can form an adhesion biofilm, characterized by the synthesis of slime on its surface [3]. Nowadays, nanomedicine has a larger number of applications, such as disease diagnosis and therapy [4,5,6]. While daptomycin and vancomycin are effective drugs against MRSA, the emergence of daptomycin-resistant strains and vancomycin-resistant S. aureus over the last decade is concerning [7,8]. Many plant extracts have potent bactericidal activity against a broad-spectrum bacterium. Plant extracts can be classified into flavonoid compounds such as tannin and flavonol, as well as non-flavonoids like phenolic acid and neolignane [9,10]. Licorice, belonging to the Leguminosae perennial family, is native to the Mediterranean region, northern China, and America [11]. Glycyrrhizic acid (GA) is one of the traditional Chinese medicines (TCMs) that is used in the treatment of many diseases and is renowned for its antitumor and antiviral effects. It is also known as glycyrrhizin, which is considered a common component in the Chinese herb licorice [12]. Previous studies have shown GA’s efficacy as an anti-allergic and anti-peptic ulcer agent [13]. GA bears structural similarity to glycyrrhetinic acid and cortisone, which exerts a strong anti-inflammatory effect. In Japan, mono-ammonium glycyrrhizinate has been used in the treatment of chronic hepatitis. Furthermore, GA has antibacterial effects against several bacterial strains [14,15,16]. Licorice has been utilized in phytomedicinal therapy for viral hepatitis, and GA has antiviral activity against several viruses, including SARS-related coronaviruses [17]. In addition, natural products, such as black pepper extract and grapefruit seed extract, can work as natural antibiotics and can inhibit multidrug-resistant pathogens. Scientists focus on natural products due to their enhancement of flavonoids like naringin [18]. Penicillin-binding proteins play multiple roles in protein’s transpeptidase (TPase) domain, contributing to the survival and growth of MRSA bacteria. The main function of PBP2 in MRSA strains is interchanged with that of PBP2A, which serves as a surrogate transpeptidase [19]. During this study, we prepared GA nanoparticles (GA-NPs) using the hydrothermal method and characterized them using physiochemical techniques. Subsequently, we investigated the antibacterial mechanism of GA-NPs against *S. aureus* and MRSA at the molecular level. Additionally, genes such as *mecA*, *mecR1*, *blaR1*, and *blaZ* were measured using an RT-qPCR assay, while the expression level of PBP2a protein in MRSA was analyzed by immunoblotting.

## 2. Results

### 2.1. Preparation and Characterization of GA-NPs

**Figure 1:**
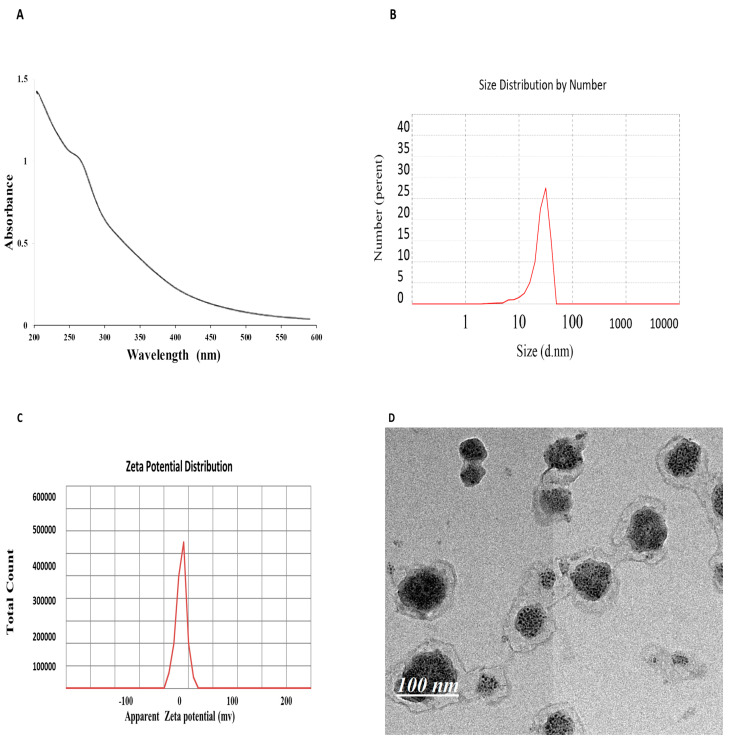
(**A**): Physical characterization of GANPs using UV–visible spectrophotometer. (**B**): DLS of GANPs. (**C**): Zeta potential of GANPs (**D**): TEM of GANPs.

**Figure 2:**
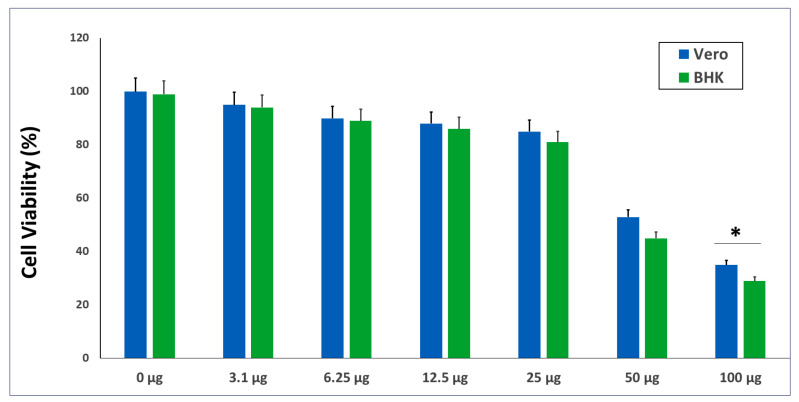
The cytotoxicity of GA-NPs against Vero and BHK cell lines. The data are presented as the mean ± standard deviation of the three independent experiments, * *p* < 0.05 vs. NC group.

The UV-vis spectrum of glycyrrhizic acid nanoparticles (GA-NPs) exhibits a minor peak at 267 nm, indicating the formation of GA-NPs (Figure 1). A dynamic light scattering (DLS) analysis of GA-NPs revealed an average particle size of 50 nm (Figure 2). Figure 1C illustrates the average ζ potential measurements, with a polydispersity index (PDI) of 0.35, indicating that GA-NPs have a negative charge of −35 mV. Finally, transmission electron microscopy confirmed the successful fabrication of GA-NPs, displaying uniform particles with well-defined distribution around 50 nm (Figure 1D).

### 2.2. The In Vitro Cytotoxicity of the GANPs

Figure 2 demonstrates the biocompatibility of GA-NPs against normal cell lines (VERO and BHK). The survival rate of the cell line stayed at approximately 100% at low concentrations of GA-NPs (3.1 and 6.25 µg/mL) but decreased to below 90% at concentrations of 12.5 and 25 µg/mL. At higher concentrations of GA-NPs (50 µg/mL), the survival rate of the cell lines dropped dramatically to 53.45% for VERO and BHK, respectively. Finally, at concentrations of 100 µg/mL, the survival percentage further decreased to 35% for VERO and 29% for BHK. These results indicate that GA-NPs exhibit low toxicity against normal cell lines.

### 2.3. In Vitro Susceptibility Test

#### 2.3.1. Disk Diffusion Method

**Figure 3:**
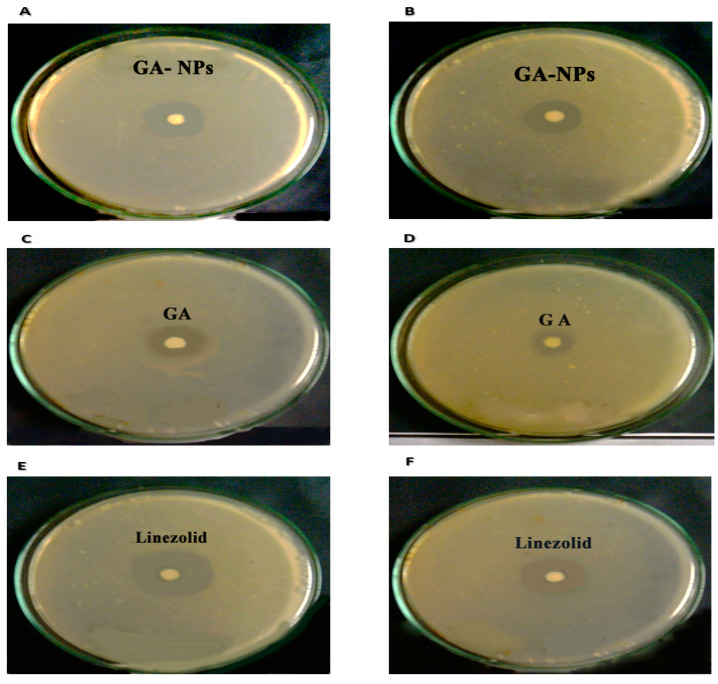
(**A**) The inhibition zone of *S. aureus* using GA-NPs. (**B**) The inhibition zone of MRSA using GA-NPs. (**C**) The inhibition zone of *S. aureus* using GA. (**D**) The inhibition zone of MRSA using GA. (**E**) The inhibition zone of *S. aureus* using linezolid. (**F**) The inhibition zone of MRSA using linezolid.

The bactericidal activity of GA-NPs against *S. aureus* and MRSA is summarized in Table 1, illustrating the effectiveness of GA-NPs for inhibiting bacterial stain activity and growth. GA-NPs subside the growth and activity of both *S*. *aureus* and MRSA. Specifically, the inhibition zones observed for *S. aureus* and MRSA after treatment with GA-NPs were 25 mm and 16 mm, respectively, as shown in Figure 3A,B.

#### 2.3.2. Minimum Inhibitory Concentration (MIC) Evaluation for Antibacterial Activity

The antimicrobial activity of GA-NPs against both bacterial strains (*S. aureus* and MRSA) is presented in Table 2, highlighting the activity of GA-NPs to suppress the growth rate and survival of the bacterial strains. The results show that GA-NPs effectively stopped the growth of *S. aureus* and MRSA at concentrations of 10.9 and 9 µg/mL for, respectively.

#### 2.3.3. Time-Kill Assay

**Figure 4:**
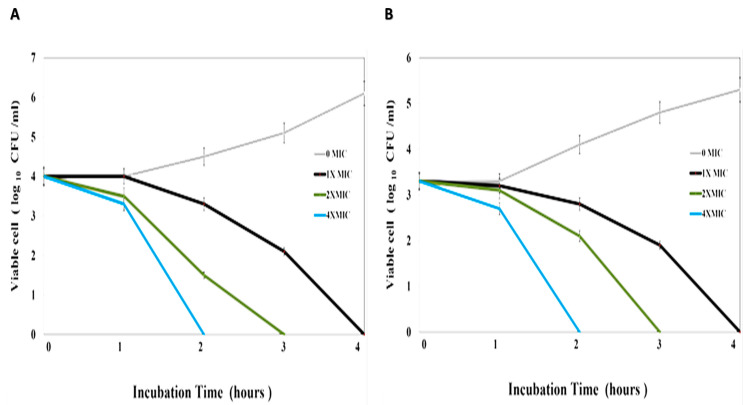
Kill-time assays: (**A**) GA-NPs against Staphylococcus aureus; (**B**) GA-NPs against MRSA. The data are presented as the mean ± standard deviation of the three independent experiments.

The antibacterial activity of GA-NPs had a strong impact on *S. aureus*. Figure 4A displays the ability of GA-NPs to decrease the quantity of bacteria in CFU/mL. Additionally, the killing time of GA-NPs against *S. aureus* occurred within 2 h of incubation at different concentrations (2× MIC: 21.6 μg/mL and 4× MIC: 43.2 μg/mL), as depicted in Figure 4A. Also, as shown in Figure 4B, the antibacterial activity of GA-NPs had a strong effect against MRSA. The killing kinetic time of GA-NPs against MRSA was achieved after 2 h of incubation at 2× MIC (18 μg/mL) and 4× MIC (36 μg/mL). These findings underscore the effectiveness of GA-NPs against *S. aureus* and MRSA bacterial strains.

#### 2.3.4. Effect of Different GA-NP Concentrations on Biofilms

**Figure 5:**
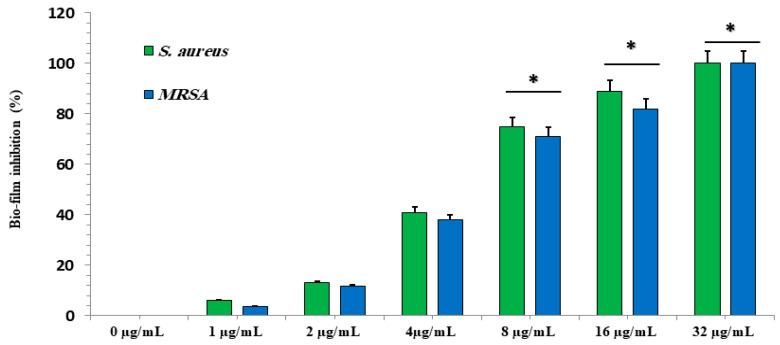
The effect of GA-NP concentrations (in μg/mL) on the viability of *S. aureus* and methicillin-sensitive *Staphylococcus aureus* biofilms compared to non-therapy with GA-NPs. The data are presented as the mean ± standard deviation of the three independent experiments, and * shows statistically significant differences at *p* < 0.05 vs. NC group.

The activity of GA-NPs at different concentrations (ranging from 1 to 32 μg/mL) aimed to restrict and stop the formation of biofilms by both *S. aureus* and MRSA. The anti-biofilm percentage of GA-NPs at 4 µg/mL was less than 20%, as Figure 5 displays. As the concentrations of GA-NPs increased, the anti-biofilm efficacy against both *S. aureus* and MRSA also increased, reaching 99.9% at concentrations of 32 μg/mL. Finally, the lowest effective concentration of GA-NPs that inhibited biofilms by 80% for both *S. aureus* and MRSA was determined to be 8 μg/mL.

#### 2.3.5. ATPase Activity Assay

**Figure 6:**
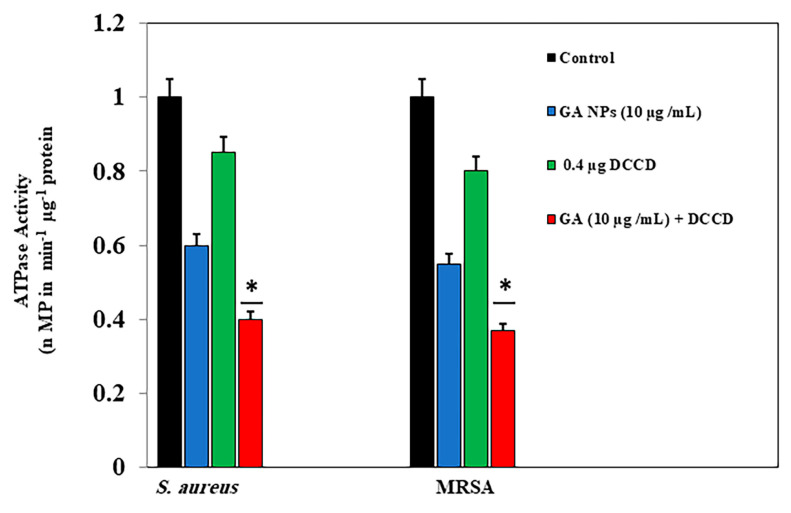
The ATPase activity of *S. aureus* and MRSA membrane vesicles in the presence of GA-NPs and DCCD (0.4 µg). The control was without NP addition. Each point represents the mean ± SD (*n* = 3). And * shows statistically significant differences at *p* < 0.05 vs. NC group.

The decreased ATP levels can be attributed to the perturbation of the electrochemical proton gradient following treatment with GA-NPs. Figure 6 demonstrates the effectiveness of the membrane-permeabilizing agent ATPase inhibitors in inhibiting the growth of both *S. aureus* and MRSA. Specifically, TX-100 facilitates the permeability of the outer membrane, particularly evident after treatment with 15 µg/mL GA-NPs associated with 0.4 mM DCCD. Under these conditions, the viability of *S. aureus* and MRSA decreased by 43.5 and 45%, respectively. As compared with the OD 600 value of 15 µg/mL GA-NPs alone, the OD 600 value of the suspension including 15 µg/mL GA-NPs is 0.00001%. TX-100 reduced *S. aureus* and MRSA by 40 and 37%, respectively. Overall, GA-NPs exhibit potent inhibitory effects on the growth of both *S. aureus* and MRSA due to their ability to affect the ATPase activity. Furthermore, the presence of an inhibitor such as DCCD, which targets the H^+^ translocator, alters ATPase activity.

#### 2.3.6. GA-NPs Represses the Transcription of *mecA*, *blaZ*, *blaR1*, and *mecR1* in *S. aureus* and MRSA

**Figure 7:**
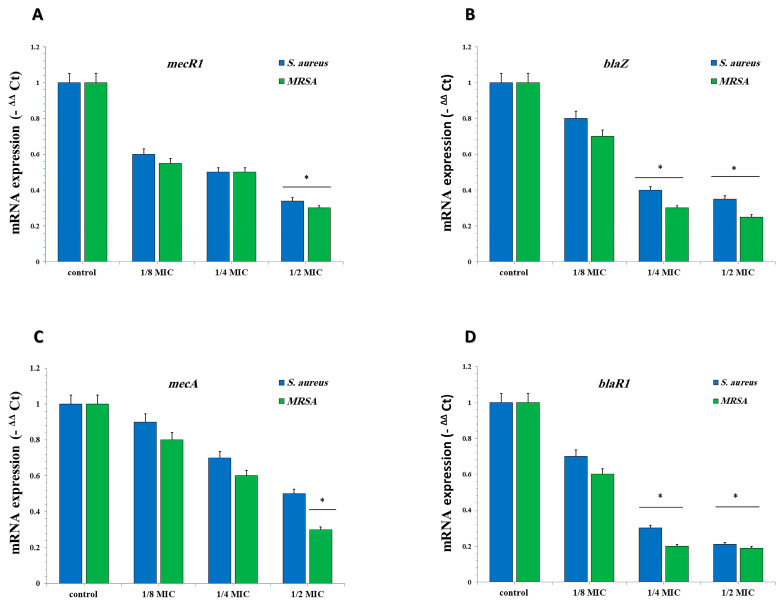
The relative gene expressions of *blaR1*, *blaZ*, *mecA*, and *mecR1* in *S. aureus* and MRSA after growth at sub-concentrations of GA-NPs. The relative gene expressions of (**A**) *blaR1*, (**B**) *blaZ*, (**C**) *mecA*, and (**D**) *mecR1* were reduced in a dose-dependent manner. The data are presented as the mean ± standard deviation of the three independent experiments. * Represents *p* < 0.05. Control, untreated control, *S. aureus*, and MRSA.

The gene expression levels of *blaZ*, *blaR1*, *mecA*, and *mecR1* were suppressed in both *S*. *aureus* and MRSA upon remediation with one-eighth MIC concentrations of GA-NPs (1.35 and 1.125 µg/mL for *S. aureus* and MRSA, respectively). Figure 7A–D illustrate the graded subinhibitory concentrations of GA-NPs and their effect on the transcription of these four genes. At one-half MIC concentrations of GA-NPs (5.4 for *S. aureus* and 4.5 µg/mL for MRSA), the transcriptional levels of *blaZ*, *blaR1*, *mecA*, and *mecR1* decreased by 1.9-fold, 2.6-fold, 2.7-fold, and 2.6-fold, respectively.

#### 2.3.7. Expression of PBP2a in *S. aureus* and MRSA

**Figure 8:**
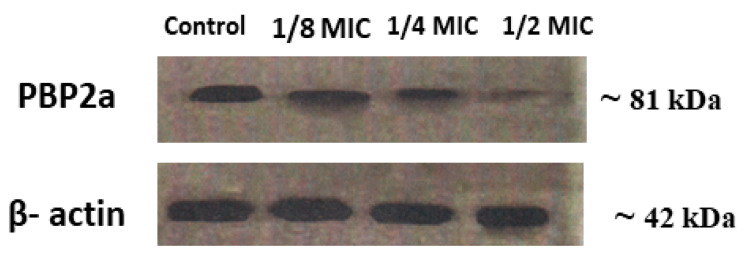
The expression of PBP2a in MRSA culture in the existence of different concentrations of GA-NPs. Each point represents the mean ± SD (*n* = 3).

The PBP2a protein expression after treatment with different concentrations of GA-NPs is displayed in Figure 8. The tested samples included a non-treated sample (Lane 1), one-eighth MIC GA-NPs (Lane 2), one-quarter MIC (Lane 3), and one-half MIC GA-NPs (Lane 4). As illustrated in Figure 8, the level of PBP2a decreased as the concentration of GA-NPs increased, especially evident with one-half MIC GA-NPs (4.5 µg/mL). A marked decrease in PBP2a expression was observed. These results demonstrated that the increase in GA-NPs led to a dose-dependent reduction in protein expression.

## 3. Discussion

*Staphylococcus* species represent a global health risk due to their ability to cause human infections such as wound infections and septicemia. Additionally, *Staphylococcus* species are implicated in several diseases such as endocarditis, osteomyelitis, and pneumonia [20]. Methicillin-resistant *S. aureus* (MRSA) represents a serious clinical challenge, exhibiting significant resistance to many drugs [21]. Currently, glycopeptides like vancomycin stand as the primary therapeutic agents used to treat MRSA globally [22]. Recently, researchers have explored and evaluated a novel approach targeting antibiotic-resistant bacteria [23]. Previous studies have documented the activity of honeydew and several plant extracts, such as black pepper extract and grapefruit seed extract (GSE), to work as bactericide agents against multidrug-resistant pathogens [24]. GSE exhibits the ability to restrict and inhibit MRSA- and vancomycin-resistant *S. aureus* (VRSA) [18]. In addition, the antimicrobial activity of *Salvia* spp. has been reported, demonstrating its effectiveness in Gram-positive strains by disrupting and damaging the cellular membrane structure [25]. Generally, glycyrrhizin has been used as an anti-inflammatory agent due to its ability to decrease the generation of reactive oxygen species (ROS) in human neutrophils. Also, glycyrrhizin can work as an anticancer agent against various cancer cells, such as primary effusion lymphoma cells, through mitochondrial extrinsic pathway apoptosis [26]. In addition, glycyrrhizin can improve the susceptibility of MRSA to β-lactam antibiotics [27]. Interestingly, glycyrrhizin extract contributes to MRSA inhibition by downregulating the expression of the MSRA genes *mecA*, *mecI*, and *mecRI* [28]. Previous reports have highlighted the bactericidal activity of glycyrrhetinic acid derivatives. Long et al. documented the efficiency of GRA at a high concentration (above 62.5 mg/L) against inhibited *S. aureus* [29]. In our study, GA-NPs were synthesized using the hydrothermal method. Characterization confirmed the spherical shape of GA-NPs with a diameter of 50 nm. Zhao et al. suggested that the formation of GA-NPs depends on alkaline pH and temperature [30]. Additionally, previous work mentioned the preparation of rosmarinic acid-derived nanoparticles (RA-NPs) [31]. Furthermore, functionalized quantum dots based on GA, synthesized using a hydrothermal approach with low cytotoxicity, have been reported [32]. Cytotoxicity data have shown the biocompatibility of GA-NPs with normal cell lines. The MIC and kill-time assay results confirmed that GA-NPs have high-impact toxicity against *S. aureus* and MRSA (10.9 and 9 µg/mL, respectively) compared to GA alone (8.2 and 7.4 µg/mL, respectively). The lowest effective concentration of GA-NPs that inhibited *S. aureus* and MRSA and biofilms by more than 80% was determined to be 8 g/mL. Similar to GA-NPs, GA-NPs exhibit potent bactericidal activity against both *S. aureus* and MRSA by impacting ATPase. Additionally, in the absence of an inhibitor such as the H+-translocating enzyme DCCD, ATPase activity was modulated. The antimicrobial activity of GA-NPs at different concentrations led to the downregulation of the *mecR1*, *blaR1*, *mecA*, and *blaZ* genes in a concentration-dependent manner. PBP2a, encoded by the mecA gene, plays a pivotal role in MRSA resistance to beta-lactam antibiotics [33]. Importantly, the penicillin-binding protein has a central role in the transpeptidase (TPase) domain of the protein, which contributes to the survival and growth of MRSA bacteria. The PBP2 function of MRSA strains is replaced by PBP2A. It serves as a surrogate transpeptidase [34]. The only method to confirm the activity of TPase in PBP2 depends on sequence homology with established transpeptidases [35]. Previous studies reported the ability of ceftizoxime to inhibit PBPs in *S. aureus*. Our results are consistent with these reports, indicating an IC_50_ of ceftizoxime against PBP2 in *S. aureus* of 0.0626 μg/mL [36]. PBP2 contributes to the production of peptidoglycan in the cell wall by activating TPase, which enriches the formation of uncross-linked muropeptide monomers [34]. Overall, our findings suggest that GA-NPs may serve as a novel, effective, and low-toxicity therapeutic agent for the treatment of MRSA.

## 4. Materials and Methods

### 4.1. Synthesis and Characterization

GA nanoparticles are prepared using a hydrothermal method. According to Zhao et al. (2021) [30], in detail, GA (15 mg/mL), which was obtained from Aldrich, Burlington, MA, USA, was dissolved in ultrapure distilled water at pH = 9.0 by using NH_4_OH, and then the mixture was incubated at 180 °C for 5 h. Therefore, the sample was centrifuged at 15,000 rpm for 15 min. The supernatant was discarded, and the pellets were further removed using a dialysis bag with a molecular weight of 14 kD to dialyze deionized water for 12 h. During the dialysis process, the DW was changed every 2 h to obtain GA-NP powder, which was then collected and freeze-dried for further use.

### 4.2. Characterization

The prepared GA-NPs were characterized by UV-Vis absorption spectroscopy (Evolution 300 UV-Vis Spectrophotometer, Thermo Scientific, Waltham, MA, USA). The size of GA-NPs in a cell culture medium was determined by dynamic light scattering (DLS) (Nano-ZetaSizer-HT, Malvern Instruments, Malvern, UK). The morphology of GA-NPs was elucidated by using high-resolution transmission electron microscopy (HRTEM; JSM-2100F, JEOL Inc., Tokyo, Japan) at an accelerating voltage of 15 kV and 200 kV.

### 4.3. The Viability of GA-NPs

The cytotoxicity of GA-NPs was determined by applying an MTT assay to VERO (African green monkey kidney epithelial cells) and BHK (Baby Hamster Kidney Fibroblasts) cell lines [5]. A total of 3 × 10^4^ cells were plated in 96-well plates and inoculated in 100 µL of growth media (DMEM medium). Then, incubated at RT overnight, the cell was then remedied with different concentrations of GA-NPs for 24 h. Then, the medium was discarded and replaced with MTT solution, dissolved in 0.5 mg/mL of MTT solution in 10% of the culture volume, and incubated at RT for 4 h till the formazan color appeared. Then, formazan was dissolved in acidified isopropanol and centrifuged at 2500× *g* for 10 min, the supernatant was transferred to new wells, and the absorbance was measured at 570 nm by a microplate reader (ELX-800 n, BioTek, Shoreline, WA, USA).

### 4.4. Bacteria Strain Preparation

In the present study, two bacterial strains were tested against GA-NPs. Various strains, such as *S. aureus* ATCC 25923 and MRSA ATCC 33591, were obtained from the ATCC (Rockville, MD, USA). The bacterial strains were cultured in Mueller–Hinton broth (MHB) (Merck, Darmstadt, Germany) in standard conditions at 37 °C for 24 h with 200 rpm agitation.

### 4.5. In Vitro Susceptibility Test

#### 4.5.1. Disk Diffusion Method

According to Sharaf et al. (2022), the bactericide activity of both GA-NPs was tested against *S. aureus* and MRSA [3]. In brief, the mentioned bacterial strains were spread on MHB plates. Then, all the testing material (GA-NPs, GA, and linezolid) was loaded with 30 μL at a concentration of 25 μg on the paper disks, while a blank disk worked as a negative control. Consequentially, the disks were incubated for 24 h at 37 °C. The inhibition zone was evaluated after 24 h of incubation.

#### 4.5.2. MIC Assay

According to Hassan et al. (2023) [1], MIC GA-NPs were evaluated using the broth microdilution method. Typically, a 106 CFU/ML inoculation of *S. aureus* and MRSA was applied to a 96-well microtiter plate. The bacterial inoculums were diluted twice using a 100 mL stock solution of GA-NPs (500 µM/mL) in 100 mL of MHB. After that, a resazurin solution was added to each well, and they were all incubated at 37 °C for 24 h. Should the hue shift from purple to pink, it would suggest the presence of bacteria [19].

#### 4.5.3. Time-Kill Assay

A total of 10^6^ CFU/mL of *S. aureus* and MRSA was cultivated in a microtiter plate. Then, a 100 mL stored solution of GA-NPs (500 µM/mL) was diluted with MHB media, including bacterial inoculums, to form the following concentrations (0× MIC, 1× MIC, 2× MIC, and 4× MIC) for *S. aureus* and MRSA in a total final volume of 1 mL, followed by the culture being incubated at RT with agitation speed at 150 rpm for 48 h. Then, the cultures were encumbered onto MHA plates at various time intervals (0, 30, 60, 120, and 240 min). The number of colonies on the MHA plates was determined in CFU/mL after incubation for 24 h [37].

#### 4.5.4. ATPase Activity Assay

The bactericidal activity of GA-NPs against bacterial strains (*S. aureus* and MRSA) was assessed in the presence of the ATPase inhibitor DCCD, which measured the ability of GA-NPs to cooperate with membrane function. The central concept of the test is the release of inorganic phosphate (Pi) when 3 mM ATP is added to the membrane [38]. The bacteria were tested in the presence of GA-NPs (15 μg/mL). Membrane vesicles were treated with 0.2 mM DCCD for 10 min [38,39].

#### 4.5.5. Anti-Biofilm Activity of GA-NPs

The activity of GA-NPs to work as an anti-biofilm agent was examined according to Peeters et al. (2008) [40]. The bacterial strains were diluted 1:100 in nutritional broth, and 100 µL of the diluted inoculum was placed in the rotatory shark at 70 rpm and incubated at RT for 24 h. Then, any planktonic bacteria were removed by washing them with sterile saline (0.9% *w*/*v*). Biofilms were treated with serial dilutions of GA-NPs (1–32 μg/mL), then incubated at 37 °C on a rotating machine for 24 h. The microbial viability was calculated and evaluated by using the AlamarBlue cell viability assay [20].

#### 4.5.6. Reverse Transcription qPCR

MRSA and *S. aureus* were treated with different concentrations of GA-NPs (12.5%, 25%, and 50% MIC) for 30 min; the non-treated was used as a negative control. A specific RNA extraction kit (Qiagen, Valencia, CA, USA) was used to extract total RNA. The RNA was measured using an A260 on a NanoDrop spectrophotometer (BioTek, Winooski, VT, USA). The RNA template was created through the transcription of RNA into cDNA using a cDNA synthesis kit (Qiagen, Valencia, CA, USA). The list of primer pairs used in RT-RCR is presented in Table 3. The steps of the RT-PCR process were initialized by adding 10 µL of 2-SYBR premix (Life Technologies, Carlsbad, CA, USA), 2 µL of sample cDNA, 1 µL of each primer (10 µM), and 20 µL of DW. The PCR was run with the Step One Plus real-time PCR system (Applied Biosystems, Foster City, CA, USA) [21].

#### 4.5.7. Western Blot Analysis

MSRA proteins were isolated using an extraction kit (iNtRON Biotechnology, Kirkland, WA, USA), which included Tris-HCI (pH 7.5). All of the protein concentrations were extracted using a Bio-Rad protein assay reagent (Bio-Rad Laboratories, Hercules, CA, USA). The supernatant was removed, and proteins were transferred to fresh tubes. Proteins were separated utilizing SDS-PAGE and subsequently transferred onto nitrocellulose membranes (Millipore, MA, USA) for 3 h at 250 mA at 4 °C using the Bio-Rad electroblotting system (Bio-Rad Mini Trans-Blot Electrophoretic Transfer Cell). To block all unreacted holes in the membrane, a solution consisting of 5% skim milk in Tris-buffered saline and Tween-20 buffer was applied. The membranes were then probed with monoclonal mouse anti-PBP2a primary antibody and β-actin (diluted 1:1000; Bio-Rad, USA), then re-probed with anti-mouse IgG secondary antibody (diluted 1:2000, Enzo Life Sciences, Ann Arbor, MI, USA). Following the treatment of the membranes with ECL Prime Western Blotting Detection Reagent (Invitrogen, Waltham, MA, USA), an Image Quant LAS-4000 mini chemical luminescent imager (GE Healthcare Life Sciences, Issaquah, WA, USA) was used to observe the bands [22].

#### 4.5.8. Statistical Analysis

In our work, SPSS 17 software packages (SPSS Inc., Chicago, IL, USA) were applied to carry out a statistical analysis by applying a one-way ANOVA test with *p* < 0.05, which is considered a significant value. Each experiment was carried out three times, and we measured the mean values and standard deviations [23,24].

## 5. Conclusions

In the present study, GA-NPs were synthesized using the hydrothermal method. The characterization of GA-NPs was carried out using physiochemical techniques, including UV–visible, DLS, and TEM. Subsequently, the antimicrobial toxicity efficiency was assessed through multiple assays. The molecular mechanism of the interaction of GA-NPs against bacterial strains was studied using RT-PCR and immunoblotting assays. The results demonstrated the successful fabrication of spherical glycyrrhizic acid nanoparticles with a diameter ranging around 50 nm. Notably, the preparation process ensured the formation of glycyrrhizic acid nanoparticles in a spherical form without any aggregation. The results displayed that glycyrrhizic acid nanoparticles have promising bactericidal effectiveness against *S. aureus* and MRSA. The results revealed a significant decrease in the transcription genes and the PBP2a protein expression in both *S. aureus* and MRSA after exposure to GA-NPs. Overall, these findings support the promising antimicrobial effect of GA-NPs and suggest that they may serve as a novel, effective, and low-toxicity therapeutic agent for the treatment of MRSA.

## Figures and Tables

**Table 1:** The inhibition zone (mm), for GA-NPs, GA, and linezolid.

Test Material	Inhibition Zone of *S. aureus*	Inhibition Zone of MRSA
GA-NPs	25 ± 0.04	16 ± 0.1
GA	16 ± 0.02	13 ± 0.05
Linezolid (LZD)	35 ± 0.01	23 ± 0.12

The data are presented as the mean ± standard deviation of the three independent experiments.

**Table 2:** The MIC value (µg/mL) for GA-NPs.

Test Material	MIC (µg/mL)	
	*S. aureus*	MRSA
GA-NPs	10.9 ± 0.01	9 ± 0.01
GA	13.9 ± 0.08	12 ± 0.03
Linezolid (LZD)	8.2 ± 0.01	7.4 ± 0.01

The data are presented as the mean ± standard deviation of the three independent experiments.

**Table 3:** The list of primer pairs applied in the qRT-PCR.

Primer	Sequence (5′-3′)
*16S RNA*	F:ACTCCTACGGGAGGCAGCAG
	R:ATTACCGCGGCTGCTGG
*mecA*	F:CAATGCCAAAATCTCAGGTAAAGTG
	R:AACCATCGTTACGGATTGCTTC
*mecR1*	F:GTGCTCGTCTCCACGTTAATTCCA
	R:GACTAACCGAAGAAGTCGTGTCAG
*blaR1*	F:CACTATTCTCAGAATGACTTGGT
	R:GACTAACCGAAGAAGTCGTGTCAG
*blaZ*	F:GCTTTAAAAGAACTTATTGAGGCTTC
	R:CCACCGATYTCKTTTATAATTT

## Data Availability

The data presented in this study are available. The original contributions presented in the study are included in the article/Supplementary Materials; further inquiries can be directed to the corresponding author.

## Supplementary Materials

The following supporting information can be downloaded at: https://www.mdpi.com/article/10.3390/ph17050589/s1, Supplemental tables concerned with the statistaical analysis of the Rt-PCR results.
