# Supplementary material for: Glycyrrhizic Acid Nanoparticles Subside the Activity of Methicillin-Resistant Staphylococcus aureus by Suppressing PBP2a
Source: Pharmaceuticals (Basel). 2024 May 6;17(5):589. doi: 10.3390/ph17050589 (PMC11123903; doi:10.3390/ph17050589)
Supplement: Supplementary file 1 [file pharmaceuticals-17-00589-s001.zip › pharmaceuticals-2940977-supplementary.pdf]

Table S1: Relative expression of *mecR1 in S. aureus* & *MRSA*

genes	conditions	expression fold changes	standard deviation	student t-tails	P-values
mecR1 S. aureus	**Control**	1.0000	0		
	**1/8 MIC**	0.62130	0.348110	0.045633735	< **0.05**
	**¼ MIC**	0.51237	0.467902	0.0310790542	< **0.05**
	**½ MIC**	0.34278*	1.153423	0.0213530425	< **0.05**
mecR1 MRSA	**Negative Control**	1.000	0		
	**1/8 MIC**	0.5678911	0.98367	0.020255842	**<0.05**
	**¼ MIC**	0.4997364	0.2539	0.057644351	**<0.05**
	**½ MIC**	0.312578*	1.8593	0.023103282	**<0.05**

Table S2: Relative expression of *blaZ in S. aureus* & *MRSA*

genes	conditions	Expression fold changes	standard deviation	student t-tails	P-values
blaZ S. aureus	**Control**	1.0000	0		
	**1/8 MIC**	0.8168991	0.257102	0.036643835	< **0.05**
	**¼ MIC**	0.402371	0.20679	0.0210790542	< **0.05**
	**½ MIC**	0.35277*	1.1624	0.0214530425	< **0.05**
BlaZ MRSA	**Negative Control**	1.000	0		
	**1/8 MIC**	0.715679	0.88357	0.020305942	**<0.05**
	**¼ MIC**	0.314734	0.2439	0.047534351	**<0.05**
	**½ MIC**	0.249578*	1.9584	0.032103282	**<0.05**

Table S3: Relative expression of *mecA* in *S. aureus* & *MRSA*

genes	conditions	Expression fold changes	standard deviation	student t-tails	P-values
mecA S. aureus	**Control**	1.0000	0		
	**1/8 MIC**	0.9116978	0.118102	0.032147514	< **0.05**
	**¼ MIC**	0.701591	0.23870	0.0120680431	< **0.05**
	**½ MIC**	0.52367*	1.4624	0.0274622136	< **0.05**
mecA MRSA	**Negative Control**	1.000	0		
	**1/8 MIC**	0.803088	0.79224	0.019424831	**<0.05**
	**¼ MIC**	0.5997412	0.29813	0.036433480	**<0.05**
	**½ MIC**	0.298457*	1.9547	0.023224363	**<0.05**

Table S4: Relative expression of *blaR1* in *S. aureus* & *MRSA*

genes	conditions	Expression fold changes	standard deviation	student t-tails	P-values
blaR1 S. aureus	**Control**	1.0000	0		
	**1/8 MIC**	0.7006872	0.198223	0.0266438351	< **0.05**
	**¼ MIC**	0.3124392*	1.912596	0.0191870634	< **0.05**
	**½ MIC**	0.210361*	1.2366	0.0332602018	< **0.05**
blaR1 MRSA	**Negative Control**	1.000	0		
	**1/8 MIC**	0.6033482	0.423604	0.044652721	**<0.05**
	**¼ MIC**	0.237661*	1.4206	0.022105576	**<0.05**
	**½ MIC**	0.1897649*	1.7949	0.024361938	**<0.05**
